# The Influence of Monomer Structure on the Properties of Ionogels Obtained by Thiol–Ene Photopolymerization

**DOI:** 10.3390/gels7040214

**Published:** 2021-11-15

**Authors:** Aneta Lewandowska, Piotr Gajewski, Katarzyna Szcześniak, Agnieszka Marcinkowska

**Affiliations:** Institute of Chemical Technology and Engineering, Poznan University of Technology, Berdychowo 4, 60-965 Poznan, Poland; katarzyna.szczesniak@put.poznan.pl (K.S.); agnieszka.marcinkowska@put.poznan.pl (A.M.)

**Keywords:** photopolymerization kinetics, thiol–ene polymerization, ionogels, gel polymer electrolytes, Kamlet–Taft parameters

## Abstract

The influence of ene and thiol monomer structure on the mechanical and electrochemical properties of thiol–ene polymeric ionogels were investigated. Ionogels were obtained in situ by thiol–ene photopolymerization of 1,3,5-triallyl-1,3,5-triazine-2,4,6(1H,3H,5H)-trione (TATT), 2,4,6-triallyloxy-1,3,5-triazine (TAT), diallyl phthalate (DAP), and glyoxal bis(diallyl acetal) (GBDA) used as enes and trimethylolpropane tris(3-mercaptopropionate) (TMPTP), pentaerythritol tetrakis(3-mercaptopropionate) (PETMP), and pentaerythritol tetrakis(3-mercaptobutyrate) (PETMB) used as thiols in 70 wt.% of ionic liquid 1-ethyl-3-methylimidazolium bis(trifluoromethylsulfonyl)imide (EMImNTf_2_). The mechanical strength of ionogels was studied by puncture resistance and ionic conductivity by electrochemical impedance spectroscopy. The course of photopolymerization by photo-DSC method (differential scanning calorimetry) as well as characterization of compositions and its components (by IR and UV spectroscopy-Kamlet–Taft parameters) were also studied. The resulting ionogels were opaque, with phase separation, which resulted from the dispersion mechanism of polymerization. The mechanical and conductive properties of the obtained materials were found to be largely dependent on the monomer structure. Ionogels based on triazine monomers TAT and TATT were characterized by higher mechanical strength, while those based on aliphatic GBDA had the highest conductivity. These parameters are strongly related to the structure of the polymer matrix, which is in the form of connected spheres. The conductivity of ionogels was high, in the range of 3.5–5.1 mS∙cm^−1^.

## 1. Introduction

Polymeric ionogels are materials that can essentially be thought of as a three-dimensional polymeric network that percolates throughout ionic liquid (IL) and is responsible for the solid-like behavior of this material. Ionic liquid prevents a matrix from collapsing into compact mass while the polymeric matrix prevents the ionic liquid from flowing out [1]. ILs are organic salts having a melting point less than 100 °C. They consist only of ions and have many interesting properties such as negligible vapor pressure, non-flammability, a wide electrochemical window, high thermal stability, and high ionic conductivity. Due to these unique physicochemical properties, they have attracted remarkable interest by the current demand in advanced electrochemical devices, such as actuators, lithium ionic batteries, electric double-layer capacitors, dye-sensitized solar cells, or fuel cells [2,3].

Ionogels exhibit superior physical and electrochemical properties by combining the mechanical flexibility of a polymer matrix and the characteristic conductivity of ILs. In the literature, several strategies for preparing ionogels can be found. They can be obtained by (i) swelling a polymer in an IL, (ii) mixing the polymer and the IL together with a co-solvent which is subsequently removed, or (iii) polymerization of monomers in an IL, which is used as a solvent [2]. Doping methods do not allow control of the structure of the polymer used (only commercially available polymers can be used). Additionally, the dissolution of the polymer and subsequent evaporation of the solvent is highly time and energy consuming process, placing high limitations on the commercial application of this process [4]. Direct polymerization of monomers dissolved in ILs seems the most advantageous approach, thus avoiding the use of additional solvents. In addition, using monomers with various structures allows for the precise design of the matrix’s final properties. Due to the method of cross-linking, they can be divided into physical and chemical gels [2]. The first group is characterized by the presence of weak, reversible interactions, such as hydrogen bonds. In the case of polymeric gels, they are in the form of a paste or jelly with rather poor mechanical properties. The second group are chemical gels, where cross-linking is the result of a chemical reaction, so strong covalent bonds are present here. As a result, chemical ionogels have good mechanical properties, which largely depend on the chemical composition of the components (polymer, monomers). Thus, ionogels can be a fairly rigid solid or soft material.

Polymer ionogels with three-dimensional networks are an important group of gel polymer electrolytes (GPEs). They exhibit dimensional stability as well as mechanical compliance over the linear polymer-based gel electrolytes. However, the higher mechanical strength of the polymer network may be a disadvantage of these materials as it may lead to a reduction in the mobility of IL ions. This causes a significant reduction in conductivity and thus limits their electrochemical performance. Therefore, obtaining materials with good mechanical and conductive properties is a challenge. The ion transport in gel electrolytes is unique and is determined by both ions of electrolyte (IL) confined inside the polymer network and the polymer dynamics. Thus, the ion transport characteristics in gel polymer electrolytes are typically governed by the physico-chemical properties of the polymer matrix and by the ion–polymer interactions [5].

As mentioned above, one of the ways to obtain ionogels is monomer polymerization in ionic liquid. Our research group conducted research on ionogels synthesized by the thiol–ene photopolymerization method. The choice of this method allowed us to use its main advantages in the synthesis of gel polymer electrolytes [6,7]. By initiating the polymerization reaction with UV radiation, it is possible to carry out the process in a short time (in the order of minutes) and at room temperature, and therefore with low energy consumption. Moreover, it did not require the use of additional solvents in the synthesis process. This allows the photopolymerization method to be classified as environmentally friendly. The main advantages of thiol–ene photopolymerization are no oxygen inhibition, formation of a homogeneous network, and no or low shrinkage stress. The thiol–ene polymerization is a radical reaction that proceeds via a step-growth mechanism. It takes place between multifunctional thiols and multifunctional enes. The reaction is based on the addition of a thiol functional group to an ene functional group. The reaction proceeds via the addition of a thiyl radical through the ene functional group to form a carbon radical (step 1). Chain transfer of the carbon radical to another thiol functional group follows the addition step, regenerating the thiyl radical and forming the thiol–ene addition product (step 2). These successive two-step propagation reactions serve as the basis for thiol–ene polymerization [8]. The structure of thiols and enes influences the kinetics of polymerization as well as the structure and properties of the obtained polymers. Such studies were mainly carried out in bulk polymerization [9,10,11,12]. As Hoyle et al. reported in their work [12], using ene monomers with lower functionality as well as with a flexible ether group (tri(ethylene glycol) divinyl ether) enables more flexible polymer networks in contrast to using ene monomers with a rigid ring structure (triallyl-1,3,5-triazine-2,4,6(1*H*,3*H*,5*H*)-trione) and higher functionality, in polymerization with both the primary and secondary tetrathiols. In other work, information can be found about structural influence of alkenes on the mechanism and kinetics of thiol–alkene photoinitiated polymerization [11]. The main conclusion of this work is that terminal enes react very rapidly with thiol and follow the basic thiol–ene two-step reaction mechanism. Internal cis enes are characterized by an insertion–isomerization–elimination reaction series, which results in trans-ene formation. The reactivity of terminal enes is affected by substituents directly bonded to the ene. These substituents affect both the radical stability and steric hindrance. Moreover, multifunctional thiol–ene photopolymerization proceeds according to the reaction mechanism established for model systems. In paper [10], the authors present the influence of thiol structure on thiol–ene polymerization. They used three difunctional thiols with the cyclohexane ring, benzene ring, and linear structure. A thiol with a cyclohexane ring has the lowest reactivity with an alkene with a high electron density (vinyl ether), electron withdrawing alkene (methacrylate), and intermediated electron density alkene (allyl ether) compared to the planar benzene ring and linear structure. Therefore, it was proposed that the steric hindrance of the cyclohexane ring structure was the cause of the slowing down of the reaction. Of the three types of alkenes used, vinyl ether exhibited the highest reactivity with all thiols, which was attributed to a high electron density of the alkene.

However, in the case of ionogels, the polymerization takes place in an ionic liquid, and may take the form of dispersion polymerization. The ionic liquid will influence the kinetics of the process and the structure of the polymer, and thus the properties of the obtained ionogels. Not only the structure of the polymer matrix but also the interactions between the ionic liquid and the polymer matrix are very important in such materials. Therefore, we decided to conduct a comprehensive investigation of a series of thiol–ene ionogels based on five different enes and both primary and secondary thiols in one ionic liquid 1-ethyl-3-methylimidazolium bis(trifluoromethylsulfonyl)imide (EMImNTf_2_). The photopolymerization of these compositions, and the mechanical and electrochemical properties of the resultant thiol–ene ionogels, have been thoroughly characterized and analyzed.

## 2. Results and Discussion

### 2.1. Characterization of Components and Compositions

**Figure 1:**
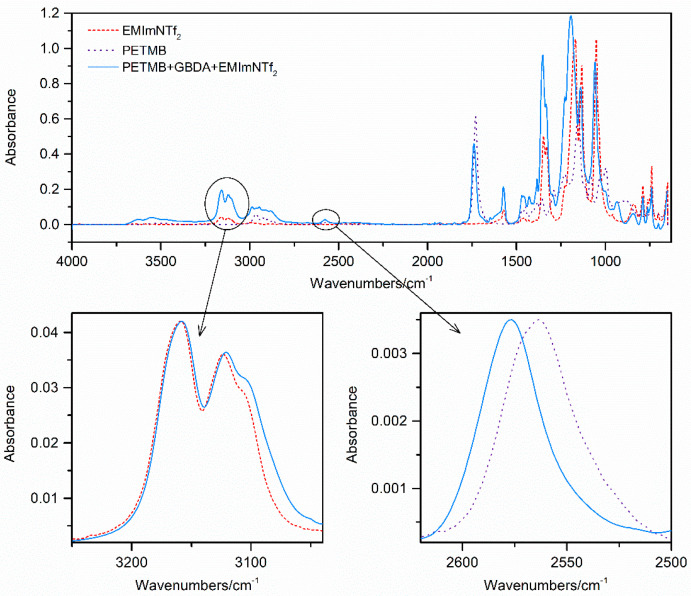
FTIR spectra of pure thiol PETMB, pure ionic liquid EMImNTf_2_, and a mixture of PETMB with ene GBDA and ionic liquid with presented shifts of absorption bands of the SH group of the thiol and C2–H of the imidazole ring. The absorbance of the spectra has been normalized for shift calculations of the studied SH and C2–H bands.

Investigations of the intermolecular interactions were carried out using the FTIR method. The interactions were observed for a given thiol–ene pair in two-component thiol+ene and three-component thiol+ene+IL systems. The results of the shifts of the absorption of SH group in thiol and C2–H of imidazolium ring bands in the mixture in relation to the pure compounds are shown in Figure 1 and Table 1.

In the case of the thiols used for research, i.e., mercaptopropionates, high polymerization rates are observed [11,13,14]. This is explained by the formation of hydrogen bonds between the thiol group and the carbonyl oxygen, which weakens the S–H bond. Additionally, in the case of TMPTP and PETMP monomers, such interactions lead to the formation of a cyclic intermediate structure of the six-membered ring, which is particularly geometrically advantageous [10,11,14]. The maximum of the absorption band of the SH group of the TMPTP and PETMP monomers occurs at the same wavenumber, i.e., 2569 cm^−1^, while in the case of the secondary thiol it is slightly shifted towards lower wavenumbers (2564 cm^−1^). The addition of GBDA ene practically does not affect the position of the absorption band of the SH group in the studied thiols. This proves that the hydrogen bonds in thiols between the SH and C=O groups are not destroyed after the introduction of ether tetraene (SH groups show little tendency to form hydrogen bonds with ether groups [15]). The addition of the remaining enes shifts the absorption band towards a higher wavenumber, with a stronger influence of the ester monomer DAP than the triazine isomeric monomers TAT and TATT. Therefore, they have a stronger impact on the destruction of hydrogen bonds between the SH and C=O groups in thiols. In three-component systems containing 70 wt.% of IL, shifts are similar in all tested systems regardless of the type of ene used. The results also indicate a slightly stronger interaction of GBDA monomer with the ionic liquid. All systems containing this monomer have a C2–H imidazolium ring bond shift greater than 2 cm^−1^. For the other systems, it remains at a similar level below 2 cm^−1^.

As we showed in our previous work [16], the course of thiol–ene polymerization in the solvent is influenced by its polarity, which can be determined by Kamlet–Taft parameters. As part of this study, we used one type of ionic liquid, so we decided to check whether the polymerization was influenced by the change in the polarity of monomers after the introduction of IL to the composition. We determined Kamlet–Taft parameters for the tested monomers, except for GBDA and TAT monomers, for which it was impossible to determine them (due to the color of the compound or high melting point). The three independent empirical Kamlet–Taft polarity parameters describe the hydrogen bond donating (α), the hydrogen bonding accepting ability (β), and the polarizability/dipolarity (π*) [17].

The obtained results are shown in Table 2. The tested monomers: thiols, enes, and ionic liquid show a similar ability to hydrogen-bonding accepting ability, while they differ in the ability to hydrogen-bond donating (α). The tested thiols are characterized by similar values of alpha and beta Kamlet–Taft parameters. They are additionally close to the Kamlet–Taft parameters of the ionic liquid EMImNTf_2_. On the other hand, enes are characterized by much lower values of the alpha parameter, which in the case of the DAP ene has the smallest value of 0.08. The TATT monomer also has slightly different characteristics, and a much higher beta value than the other compounds, which is probably due to the presence of three carbonyl oxides in the monomer molecule. Additionally, there are no hydrogen interactions in this monomer, as is the case with thiols.

### 2.2. Ionogel Synthesis

**Figure 2:**
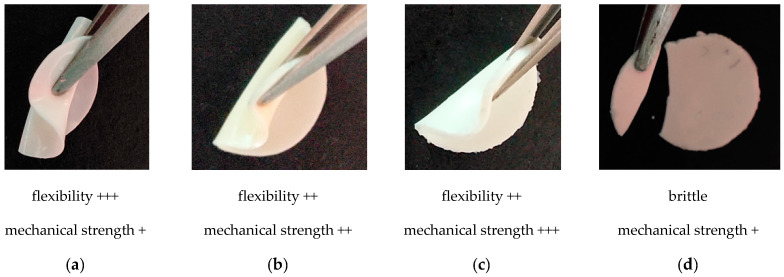
Thiol–ene ionogels obtained by polymerization of the system: (**a**) DAP + PETMB, (**b**) GBDA + PETMB, (**c**) TAT + PETMB, (**d**) TATT + PETMB. The puncture-resistance tests results (mechanical strength) are presented in Figure 7.

The study was focused on the influence of monomer structure on the properties of thiol–ene polymer ionogels, which can be used as gel polymer electrolytes in electrochemical capacitors. The aim of the research was to search for an ionogel with good mechanical properties as well as ionic conductivity. Due to the appropriate mechanical properties during the preparation of the electrochemical capacitor and its operation, there was no fear of damaging the separator. In addition, a material with high mechanical resistance may have a small thickness. Three multifunctional thiols, one three-functional and two four-functional, primary and secondary, were selected for the study. Allyl compounds of different structures were used as enes: aliphatic GBDA, aromatic DAP, triazine TAT, and additionally an isomer of the former, TATT. The thiol–ene polymerization was carried out with equimolar ratios of the SH:CC functional groups. All compositions tested, both with and without ionic liquid, were homogeneous prior to polymerization. As a result of polymerization, transparent thiol–ene polymers and opaque, white ionogels were obtained. Figure 2 shows photos of selected ionogels prepared with PETMB thiol and studied enes, containing 70 wt.% of EMImNTf_2_. They reflect the properties of all obtained ionogels: flexible but not mechanically strong (F_max_ < 0.4 N: mechanical strength +), mechanically strong (0.4 N < F_max_ < 1.0 N: mechanical strength ++ or F_max_ > 1.0 N: mechanical strength +++) but less flexible, and brittle-destroyed when attempted to roll up. As can be seen in Table 3, most of them are quite flexible, which allows them to be rolled up without damaging them.

The lack of transparency of the materials indicates a phase separation occurring between the ionic liquid and the polymer matrix. Our previous works show that the thiol–ene polymerization process in the ionic liquid EMImNTf_2_ proceeds as dispersion polymerization [7]. Dispersion polymerization begins as a homogeneous solution of monomer and the polymer phase separates during the polymerization process.

In the initial stage, the process takes place in the continuous phase, but in the later stages mainly in monomer swollen polymer particles. It is caused by the loss of solubility by the growing polymer chain in the reaction medium due to insufficient polymer–solvent interactions and/or due to the formation of a polymer network. Coagulation of the oligomer chains continues until steric stabilization of the latex particles begins [18]. No stabilization of the resulting colloid often leads to a wide particle size distribution [19,20]. The stabilizer should have an affinity for both the reaction medium and the polymer particles. In conventional organic solvents, poly-*N*-vinylpyrrolidone (PVP) is the most commonly used stabilizer [21,22,23,24]. The sterically stabilized particles keep growing until nearly complete monomer consumption [18].

**Figure 3:**
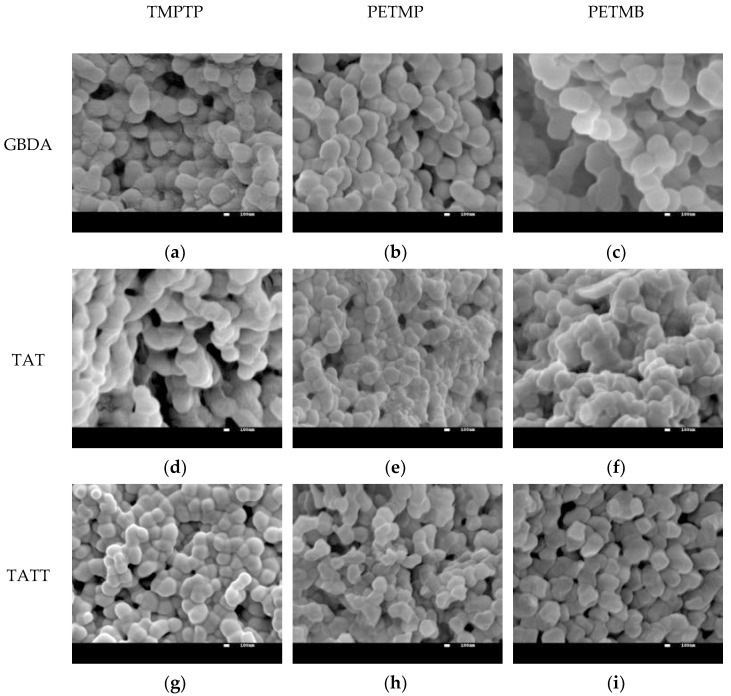

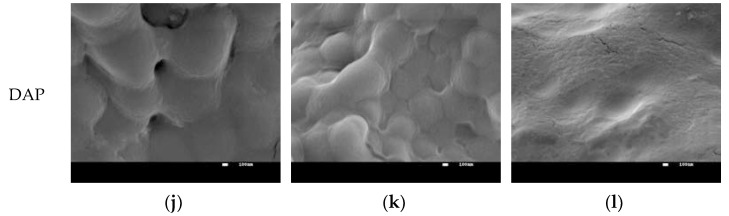
SEM pictures of polymer matrices of ionogels obtained in the study, after removing the ionic liquid. Polymer matrix obtained on the basis of different enes: (**a**–**c**) GBDA, (**d**–**f**) TAT, (**g**–**i**) TATT, and (**j**–**l**) DAP, and three different thiols: TMPTP, PETMP and PETMB.

In order to confirm whether the polymerization in the tested systems also proceeds according to the dispersion polymerization mechanism, SEM pictures of the obtained ionogels were taken (Figure 3). As can be seen, most of the polymeric ionogels matrices have the character of connected microspheres (with diameter in the range 130–430 nm). Only in the case of ionogels obtained on the basis of the DAP ene, the matrix in the form of a continuous mass with slightly marked microspheres (with diameter in the range 650–750 nm) is visible. Thus, the ionic liquid is a good solvent for monomers and a poor solvent for the polymer, causing the polymer to precipitate rapidly in the form of microspheres from the reaction mixture. At the same time, it seems that the ionic liquid has a stabilizing effect on colloidal polymer particles (e.g., high efficiency of the ionic stabilizer, the fourth-order ammonium surfactant as a dispersion polymerization stabilizer has been demonstrated [25]) thanks to which a polymer in the form of connected microspheres with a fairly uniform size was obtained. This stabilizing effect of EMImNTf_2_ in investigated thiol–ene systems is related to forming solvation shells as a result of the formation of hydrogen bonds by proton-donors and proton-acceptors, as well as through electrostatic interactions attracting counterions. Polymer particles, due to thiol structures, possess carbonyl groups on their surface which can hydrogen bond to imidazolium ionic liquid cation. This type of H-bonding will compete with cation–anion interactions and will contribute to polymer particle stabilization in IL. The weakest stabilization of the spheres occurs in the case of the polymer based on the DAP ene, which is characterized by a very low value of the Kamlet–Taft α parameter, which affects the proton-donor and proton-acceptor interactions in the system. DAP does not have the ability to give off hydrogen and therefore does not compete with the ionic liquid, which can therefore interact more strongly with the carbonyl groups of the polymer. This leads to the coagulated matrix shown in Figure 3j–l. However, the photos also show the largest spheres. As is known, the size of the microspheres depends on the solubility of the polymer in the reaction medium and on the functionality of the monomers [23,24]. The increased solubility of the polymer (greater compatibility with IL) requires a greater critical chain length or a greater degree of cross-linking for polymer nucleation to occur. The formed nuclei of the separated polymer react with the growing polymer chains and uniformly increase their size. As the functionality of the monomer increases, the solubility of the polymer decreases, nucleation begins with lower conversions, and more nuclei are formed. This in turn, for the same concentration of monomer achievable, leads to smaller microspheres. Therefore, the spheres with the largest diameter are visible in the photos of ionogels based on the DAP ene. This is related, on the one hand, to the higher compatibility of polymerizing medium with ionic liquid. On the other hand, the DAP ene monomer has the lowest functionality (F = 2) of all monomers used, so nucleation during polymerization with this monomer begins at higher conversion and thus fewer spheres are created but they grow to a larger size. In the case of using GBDA, TATT, and TAT cross-linking enes, matrices having the character of connected microspheres were obtained, and matrices with an ene GBDA which has the highest functionality (F = 4) were characterized by spheres with larger dimensions. This may indicate a slightly better compatibility of these polymer matrices with the ionic liquid than in the case of polymer matrices based on the TAT or TATT enes. An additional factor may be the greater flexibility of the network with this aliphatic monomer than with the TAT or TATT monomers with a rigid triazine core. The images of the polymer matrices obtained on the basis of the TMPTP and PETMP thiol monomer for individual enes are very similar, which may result from the analogous structure of these thiols, differing in structure with only one thiol group and the presence of the –CH_3_ group in the case of the TMPTP thiol. However, due to the fact that the equimolar ratio of the SH:CC functional groups, is used, the composition is almost identical. Some differences are evident in the case of PETMB secondary thiol, which could be due to hindered rotation of thiol–ether linkages (–S–) afforded by the additional α-methyl group of PETMB.

#### Photopolymerization Kinetics

**Figure 4:**
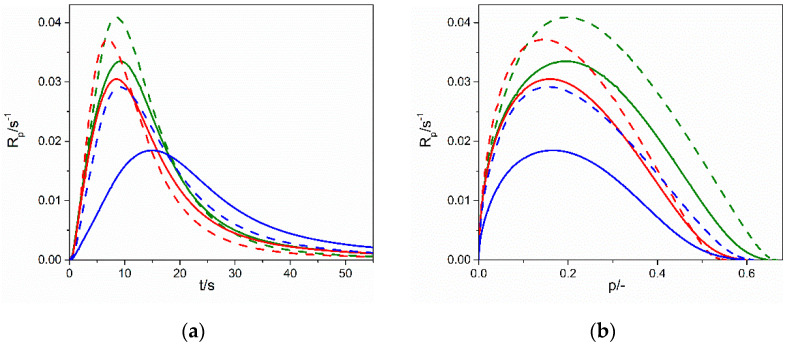

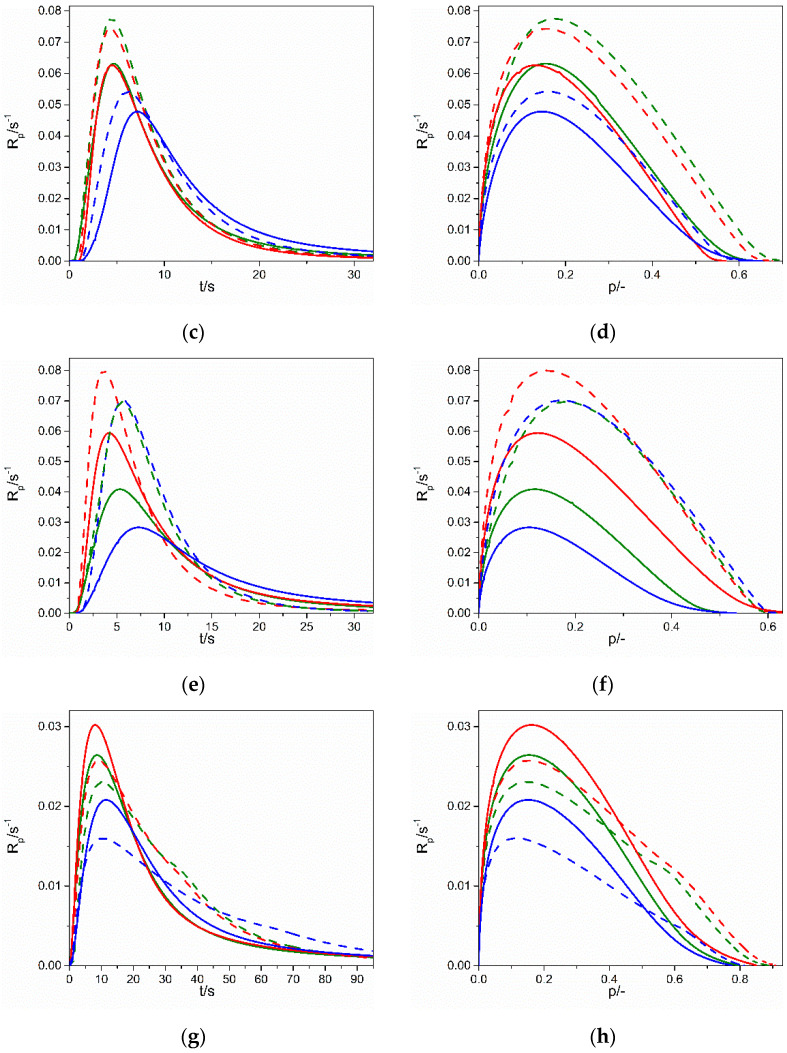
Dependence of polymerization rate (R_p_) on (**a**,**c**,**e**,**g**) polymerization time (t) and (**b**,**d**,**f**,**h**) conversion (*p*) for thiol–GBDA systems (**a**,**b**), thiol–TAT systems (**c**,**d**), thiol–TATT systems (**e**,**f**) and thiol–DAP systems (**g**,**h**) in bulk (solid lines) and in 70 wt.% of EMImNTf_2_ (dashed lines). Used thiols: TMPTP (green line), PETMP (red line), PETMB (blue line).

The polymerization kinetics of each of the thiols TMPTP, PETMP, PETMB mixed with three different types of allyl ethers, DAP, TAT, GDBA, and triazine isomer TATT, was followed by photo-DSC method. For all four types of ene monomers, as presented in Figure 4, the polymerization rate of the samples with secondary thiol, PETMB, is apparently slower: the initial and maximum polymerization rate, R_p_^max^, is lower, and also the time for reaching maximum polymerization rate, t^max^, is longer (see also Table 4 where values of R_p_^max^ and t^max^ are presented). The reaction rates in other two primary thiols are faster than for the secondary thiol, and the maximum polymerization rates in TMPTP and PETMP are similar. Ionic liquid EMImNTf_2_ accelerates the thiol–ene polymerization for all three thiols when cross-linking enes GBDA, TATT, and TAT are used. Thus, in the case of dispersion polymerization, leading to the formation of a matrix in the form of connected spheres, we observe an acceleration of the polymerization reaction in IL. It is related to the influence of the ionic liquid on the thiol–ene reaction, i.e., additional stabilization of the transition state during the chain-transfer step [6]. On the other hand, thiol–ene polymerization slows down when the linear ene DAP is used. Moreover, the kinetic curves of the DAP polymerization, especially in the polymerization with TMPTP and PETMP, show some acceleration at the end of the reaction. As can be seen from the SEM images, the thiol–DAP polymerization is only partially dispersive and partially solvent-based. It seems that the acceleration of the reaction in its final stages is related to the greater proportion of dispersion polymerization, which is manifested by more prominent spheres in the SEM images for TMPTP and PETMP enes.

**Figure 5:**
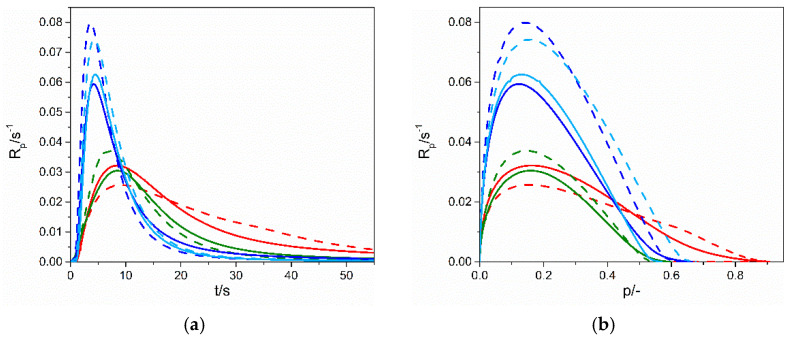
Dependence of polymerization rate (R_p_) on (**a**) polymerization time (t) and (**b**) conversion (p) for PETMP–ene systems in bulk (solid lines) and in 70 wt.% of EMImNTf_2_ (dashed lines). Used enes: DAP (red line), GBDA (green line), TAT (violet line), TATT (blue line).

**Figure 6:**
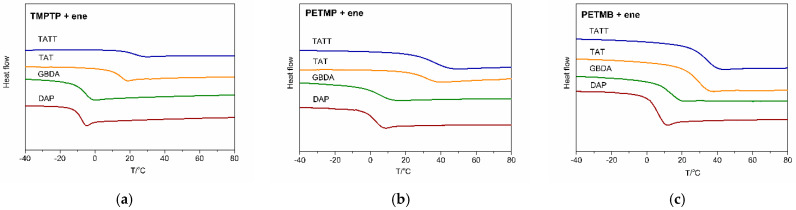
DSC curves of thiol–ene polymers obtained in bulk polymerization of the compositions: (**a**) TMPTP + ene, (**b**) PETMP + ene, (**c**) PETMB + ene. Used enes: DAP (red line), GBDA (green line), TAT (orange line), TATT (blue line).

The highest polymerization rate, both in bulk and in ionic liquid, is achieved for reaction with enes having a rigid core structure, TATT and TAT (Figure 5). The poly(PETMP-TAT) and poly(PETMP-TATT) show glass transition T_g_ (Figure 6) above room temperature (32 °C and 37 °C, respectively, Table 5), which causes additional complexity of radical trapping associated with vitrification, and thus incomplete conversion, which is about 50–60%. These high glass transition temperatures are consistent with the literature, where we can find even higher values for such polymers [12,26]. Polymerization in ionic liquid goes to almost 70% of conversion for a system with TAT and 60% of conversion for a system with TATT, which is related to the increase in the mobility of the polymer networks due to the dilution of the system. The ene with higher functionality, GBDA, polymerizes with PETMP with a lower polymerization rate, but conversion is almost the same, as in the former system. Ionic liquid accelerates the polymerization rate of this system, but the conversion is not higher, so the mobility of the network does not rise enough to increase conversion. Unfortunately, it was not possible to determine the glass transition temperature of the polymer matrix in the ionogel. On the other hand, the T_g_ of bulk polymer is 6 °C. Thus, below room temperature, as well as the earlier-mentioned T_g_ of the polymers based on TAT and TATT monomers. Thus, vitrification does not affect the polymerization process. The thiol–ene conversion of the composition with difunctional aromatic ene DAP is almost 90%, and only a slight increase is observed in the polymerization in the ionic liquid, which indicates that the increase in network mobility as a result of dilution is insignificant.

### 2.3. Mechanical and Electrochemical Properties

**Figure 7:**
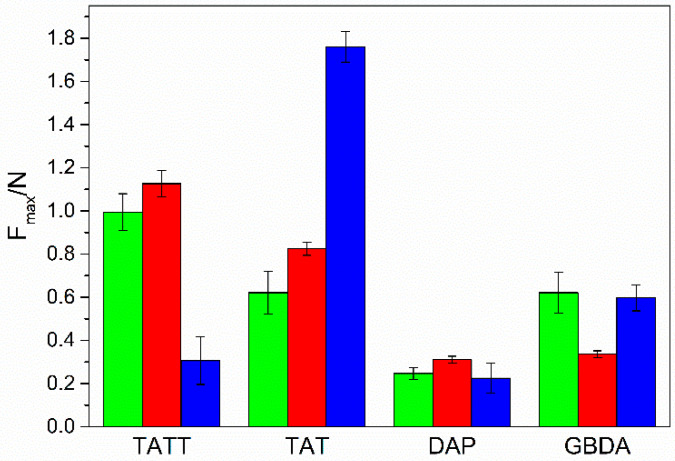
Puncture resistance for investigated ionogels obtained by photopolymerization of thiols TMPTP (green columns), PETMP (red columns), and PETMB (blue columns) with different allyl monomers: TATT, TAT, GBDA, and DAP.

Good mechanical strength of synthesized materials is a key factor for creating real application of gel polymer electrolytes. To investigate the mechanical strength of obtained ionogels, puncture-resistance tests have been performed by varying the thiol and ene monomer structures. The effect of these factors is well observed in Figure 7. The structure of ene monomers has a great influence on the mechanical strengths of ionogels. Polymeric gels with aromatic, difunctional DAP have the weakest resistance to puncture. This can be connected rather with the structure of the ionogel matrix, i.e., microspheres embedded in a continuous polymeric matrix, than polymeric network. On the other hand, due to the presence of a continuous mass of polymer, the flexibility of these ionogels is high. The other ionogels have a very similar structure of connected spheres, but a more careful analysis shows differences in the size of the spheres and the way they are connected. A rigid triazine core in two isomeric monomers contributes to slightly better mechanical strength of ionogels obtained with these monomers than the aliphatic GBDA monomer. Moreover, ionogels with primary thiols and TATT, due to carbonyl-group presence, are more resistant to puncture. However, ionogel with secondary thiol PETMB is brittle, and it breaks upon attempting to roll it up. It seems that it is correlated with very weakly connected microspheres of this ionogel, which look like glass pearls. In contrast, ionogel with the same thiol and triazine isomeric monomer TAT is characterized by the highest puncture resistance. However, the matrix morphology of this ionogel is quite different; they are connected spheres, with a significant area connecting them.

**Figure 8:**
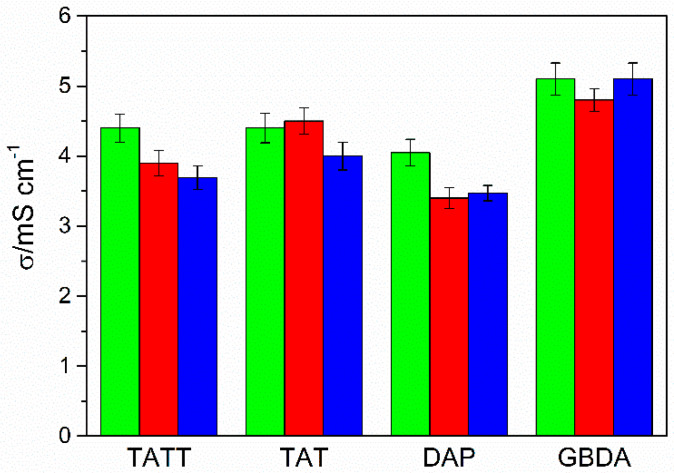
Ionic conductivity of investigated ionogels obtained by photopolymerization of thiols TMPTP (green columns), PETMP (red columns), and PETMB (blue columns) with different allyl monomers: TATT, TAT, GBDA, and DAP. RSD ≤ 5%.

Ionic conductivity of investigated ionogels (Figure 8) is in the range 3.5–5.1 mS∙cm^−1^, which accounts for 36–56% of the pure electrolyte conductivity, with the highest values obtained for ionogels synthesized on the GBDA ene. Typically, the relative conductivity for membranes with 70% electrolyte content is a maximum of 50%. Thus, gel polymer electrolytes with good conductive properties were obtained. The ionic conductivity, on the one hand, depends on the porosity of the membrane, and on the other hand, on the interactions between the electrolyte and the polymer matrix. Since all ionogels (except ionogels on the DAP basis) have porous matrices, the differences in conductivity result from interactions between the polymer and the electrolyte. Slightly larger shifts in the FTIR spectra for the C2–H band of the imidazolium ring are visible for systems with the GBDA monomer, and therefore it interacts most strongly with the ionic liquid, which may indicate a weakening of ionic interactions between ions, and thus faster diffusion of ions in the ionogel. The structure of the matrix in the case of ionogels based on the DAP ene may result in slightly lower ionic conductivity of these materials.

## 3. Conclusions

The structure of enes (TAT, TATT, GBDA, DAP) and thiols (primary TMPTP, PETMP, and secondary PETMB) affects the mechanical and conductive properties of ionogels obtained by photopolymerization of thiol–ene in the presence of an ionic liquid EMImNTf_2_. The polymerization process in the case of TAT, TATT, and GBDA enes follows the dispersion polymerization mechanism, which leads to obtaining matrices with phase separation in the form of interconnected microspheres. Their size and connection type depend on the monomer structure and affect the material properties. The larger area of connection of the spheres increases the puncture resistance of ionogels. On the other hand, the simultaneous course of the polymerization according to the dispersion and solvent mechanism, which takes place in the case of the DAP ene, leads to a mechanical weakening of the materials. These ionogels are also characterized by a slightly lower ionic conductivity. Monomer structure and functionality also influence photopolymerization kinetics. The polymerization reaction is faster in compositions with triazine isomeric monomers TAT or TATT. 

## 4. Materials and Methods

### 4.1. Materials

Monomers: 1,3,5-triallyl-1,3,5-triazine-2,4,6(1*H*,3*H*,5*H*)-trione (TATT), purity 98%, 2,4,6-triallyloxy-1,3,5-triazine (TAT), purity 97%, diallyl phthalate (DAP), purity 97%, glyoxal bis(diallyl acetal) (GBDA) purity 95%, trimethylolpropane tris(3-mercaptopropionate) (TMPTP) purity ≥ 95%, and pentaerythritol tetrakis(3-mercaptopropionate) (PETMP), purity ≥ 95% were delivered by Sigma-Aldrich (St. Louis, MO, USA) and pentaerythritol tetrakis(3-mercaptobutyrate) (PETMB), purity 97% was provided by Showa Denko KK (Tokyo, Japan). Ionic liquid (IL): 1-ethyl-3-methylimidazolium bis(trifluoromethylsulfonyl)imide (EMImNTf_2_) purity 99% was delivered by Solvionic (Toulouse, France). The photoinitiator 2,2-dimethoxy-2-phenylacetophenone (DMPA) was also supplied by Sigma-Aldrich (St. Louis, MO, USA).

### 4.2. Methods

1. Solvatochromic Solvent Parameters

Solvatochromic parameters: Reichardt’s empirical ET(30) polarity parameter, normalized polarity parameter E_T_^N^, empirical Kamlet–Taft polarity parameters: α (hydrogen bond donating ability), β (hydrogen bond accepting ability), and π* (dipolarity/polarizability) were determined using 4-nitroaniline, purity > 99%, N,*N*-diethyl-4-nitroaniline, purity 98%, and Reichardt’s dye, purity 90%. All dyes were supplied by Sigma-Aldrich (St. Louis, MO, USA). Anhydrous methanolic solutions of each dye were prepared at a concentration of 5 × 10^−3^ M. The dye solution was added to the IL and monomers, then the mixture was homogenized and the methanol was removed at 40 °C under reduced pressure. The dye concentration in each of the monomers and ionic liquid was sufficient to allow an absorbance band in the range 0.4 to 0.5. The absorption spectrum for each dye was measured by spectrophotometer Jasco UV-530 (Tokyo, Japan). All the spectroscopic measurements were carried out in the measuring range 200–800 nm in a quartz cuvette with a light path length of 1 mm at room temperature. The wavelength corresponding to the absorption maximum (λ_max_) was read from each obtained spectrum, and then the solvatochromic parameters were calculated corresponding to Equations (1)–(5) and the methods described in the articles [27,28,29,30]:

(1) $$\alpha =\frac{E_{T}\left(30\right)-14.6\left(\pi^{*}-0.23\right)-30.31}{16.5}$$

(2) $$\beta =\frac{1.035v{\left(DN\right)}_{max}-v{\left(N\right)}_{max}+2.64}{2.8}$$

(3) $$\pi^{*}=\frac{v{\left(DN\right)}_{max}-27.52}{-3.182}$$

(4) $$E_{T\left(30\right)}\;=\;\frac{28591}{\lambda {\left(RD\right)}_{max}}$$

(5) $$E_{T}^{N}=\frac{E_{T}\left(30\right)-30.7}{32.4}$$

where (DN)-*N*,*N*-diethyl-4-nitroaniline, (*N*)-4-nitroaniline, and (RD)-Reichard’s dye.

2. Ionogels Samples Synthesis

**Figure 9:**
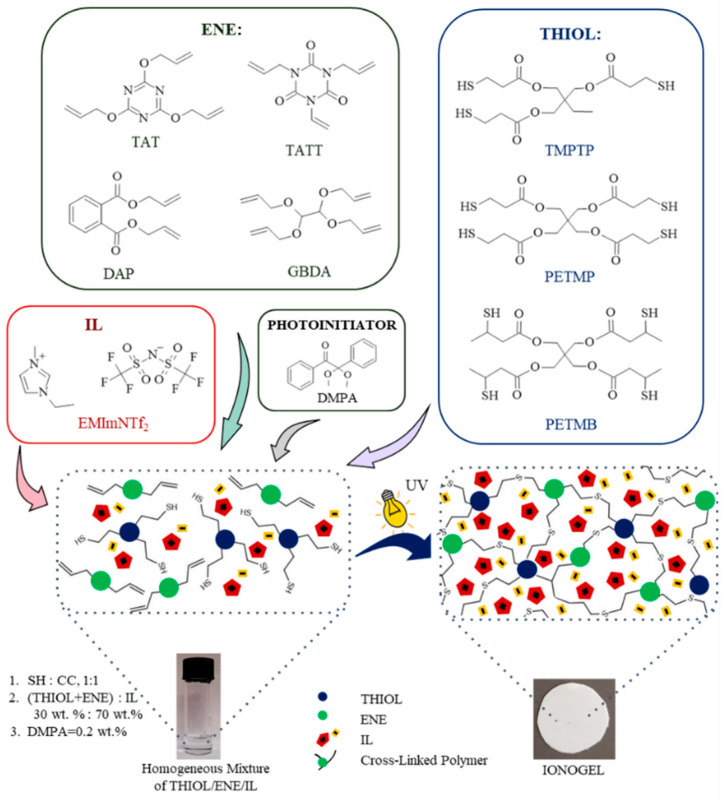
Schematic illustration of the synthesis of ionogel.

The ionogel preparation by one-pot reactions of thiols and enes with different chemical structures in the presence of an ionic liquid is shown in Figure 9. The samples were prepared in a glove box under a pure argon atmosphere. The concentration of ionic liquid in the photocurable composition was 70 wt.% calculated on the total amount of the composition. The mixture of thiol+ene monomers was used in stoichiometric ratios of ene functional groups to thiol functional groups (1:1, C=C:SH). The photoinitiator DMPA was used in concentration 0.2 wt.% calculated on the whole composition. The composition consisted of ionic liquid and a mixture of monomers, and the photoinitiator was homogenized in an orbital shaker. Obtained homogeneous photocurable composition was then poured into a glass mold with a thickness of 0.3 mm. Subsequently, the prepared composition was irradiated with UV for a proper time on two sides of the mold with ASN-36W UV lamp (λmax = 365 nm, light intensity 6 mW∙cm^−2^). For composition containing thiol TMPTP or PETMP, the duration of irradiation on one side was 5 min, and for composition with secondary thiol PETMB 10 min. Then, test samples of appropriate dimensions were cut from the obtained ionogel sheets.

3. Differential Scanning Calorimetry-DSC

Thermal transition temperatures were determined by differential scanning calorimetry (DSC) method using a DSC1 instrument (Mettler-Toledo, Greifensee, Switzerland). Measurements were performed with a heating rate of 20 °C∙min^−1^ in the temperature range from −80 °C to 180 °C under a nitrogen atmosphere. The glass transition temperatures (T_g_) were determined from the second run of the DSC measurement.

4. Isothermal Differential Scanning Photocalorimetry (Photo-DSC)

The kinetics of the thiol−ene photopolymerization in ionic liquid was performed by using isothermal differential scanning calorimetry (DSC, Pyris 6 instrument, Perkin Elmer, Waltham, MS, USA). The DSC apparatus was equipped with a lid especially designed for photopolymerization measurements. The 1.5 ± 0.2 mg samples were polymerized in open aluminum pans (diameter 6.6 mm) under isothermal conditions at 25 °C in high-purity argon (<0.0005% of O_2_) purged with 50 mL∙min^−1^ before (2 min) and during the polymerization reaction. The polymerization was initiated with UV light from an LED lamp (Hamamatsu Photonics, Hamamatsu, Japan LC-1, λ = 365 nm, I_0_ = 1 mW∙cm^−2^ at the sample pan position). All reactions were conducted at least in triplicate. The reproducibility of the kinetic results was about ±3%.

5. Puncture Resistance

The mechanical properties of the obtained ionogels were characterized by a puncture-resistance test [7]. The measurements were carried out using the CT3 texture analyzer (Ametek Brookfield, Middleboro, MA, USA). A sample with a diameter of 16 mm was cut off from the ionogel sheet immediately after synthesis. Its thickness was measured and then the sample was fixed in a 10 mm-hole diameter sample holder and tested for puncture strength using the cylindrical probe with 2.5 mm radius. During the measurement, the load and distance of the measuring probe were recorded until the sample was punctured (probe displacement rate was 0.3 mm∙s^−1^). As obtained ionogels slightly differed in their thickness, the load was normalized to a uniform thickness (300 µm) in order to compensate for the influence of thickness on the tested parameters. The study was carried out five times for each type of ionogel. Based on the obtained results, the means and standard deviations were calculated.

6. Infrared Spectroscopy

Infrared (IR) spectra were performed on a Nexus Nicolet 5700 Fourier Transform Infrared Spectrophotometer (FTIR, Thermo Electron Scientific Instruments Corporation, Madison, WI, USA) at room temperature in range 4000–600 cm^−1^ and resolution 4 cm^−1^ at 64 scans. The material tested was placed as a thin film between two NaCl plates. The IR shifts (Δ*ν*) of the C2–H bond absorption band of the imidazole ring of the IL and the SH group absorption band of the thiol group were calculated from Equation (6):

(6) $$\Delta \nu =\nu_{c}-\nu_{0}$$

where Δ*ν* is the difference between the position of the absorption peak of C2–H or SH group of investigated compounds in the photocurable composition (*ν_c_*) and pure compound (*ν_0_*).

7. Ionic Conductivity

The ionic conductivity of the ionogels was investigated by electrochemical impedance spectroscopy (EIS) using the SP-300 potentiostat/galvanostat (Biologic, Seyssinet-Pariset, France) in the frequency range from 1 kHz to 1 MHz [7]. The experiment was carried out at room temperature in a two-electrode (stainless steel—316L)-type electrochemical vessel.

The ionic conductivity of the ionogels (*σ*) was calculated from Equation (7):

(7) $$\sigma =\frac{1}{A\cdot \Delta \sigma_{s}}$$

where *σ* is the ionic conductivity of the ionogel in S∙cm^−1^, l is the thickness of the ionogel in cm, *A* is an ionogel surface area in cm^2^, and *σ_s_* represents the volumetric conductance of the ionogel sample in *S*. The residual standard deviation (RSD) for the tested samples did not exceed 5%.

In addition, the relative conductivity *σ_rel_* of investigated ionogels in relation to the conductivity of an IL, were calculated from Equation (8):

(8) $$\sigma_{rel}=\frac{\sigma }{\sigma_{IL}}$$

where *σ_IL_* is the ionic conductivity of IL in S∙cm^−1^.

## Figures and Tables

**Table 1:** Changes in the position of the absorption bands of the SH group of the thiol and C2–H of the imidazole ring of the ionic liquid in two- and three-component systems.

	TMPTP			PETMP			PETMB		
	Bulk	70 wt.% of IL		Bulk	70 wt.% of IL		Bulk	70 wt.% of IL	
	SH	SH	C2–H	SH	SH	C2-H	SH	SH	C2–H
GBDA	0.12	7.96	−2.25	0.31	8.35	−2.35	0.87	9.71	−2.12
TAT	2.56	8.04	−2.02	2.08	6.44	−1.98	2.99	10.13	−1.88
TATT	2.83	8.57	−1.72	2.87	8.36	−1.79	3.53	9.64	−1.41
DAP	3.73	8.52	−1.86	3.94	8.99	−1.72	4.71	10.36	−1.63

**Table 2:** Kamlet–Taft parameters of tested thiol and ene monomers and ionic liquid EMImNTf_2_.

Component	α	β	π*
EMImNTf_2_	0.66	0.23	0.98
TMPTP	0.56	0.33	0.87
PETMP	0.49	0.32	0.93
PETMB	0.58	0.40	0.79
DAP	0.08	0.36	0.85
TATT	0.25	0.51	0.63

**Table 3:** Flexibility of the obtained ionogels.

	Flexibility		
	TMPTP	PETMP	PETMB
GBDA	+++	+++	++
TAT	+++	++	++
TATT	+++	+	brittle
DAP	+++	+++	+++

**Table 4:** Maximum polymerization rate (R_p_^max^_,_ s^−1^) and time for reaching R_p_^max^ (t^max^, s).

	TMPTP		PETMP		PETMB	
	70 wt.% of IL					
	R_p_^max^, s^−1^	t, s	R_p_^max^, s^−1^	t, s	R_p_^max^, s^−1^	t, s
GBDA	0.041	8.5	0.037	6.9	0.029	9.2
TAT	0.077	4.4	0.074	4.3	0.054	6.1
TATT	0.070	5.6	0.080	3.6	0.070	5.6
DAP	0.023	10.3	0.026	9.1	0.016	10.2
	Bulk					
GBDA	0.034	9.2	0.031	8.5	0.018	15.1
TAT	0.063	4.7	0.062	4.5	0.048	7.1
TATT	0.041	5.3	0.059	4.2	0.028	7.3
DAP	0.026	8.6	0.031	8.0	0.021	11.4

**Table 5:** Glass transition temperature of thiol–ene polymers (obtained in bulk polymerization).

	T_g_ °C		
	TMPTP	PETMP	PETMB
GBDA	−5.7	5.9	14.2
TAT	15.3	31.7	29.6
TATT	22.9	36.6	34.3
DAP	−7.7	3.0	6.3
